# Exploring pain phenotypes in workers with chronic low back pain: Application of IMMPACT recommendations

**DOI:** 10.1080/24740527.2020.1870103

**Published:** 2021-03-03

**Authors:** Lisa C. Carlesso, Yannick Tousignant-Laflamme, William Shaw, Christian Larivière, Manon Choinière

**Affiliations:** a School of Rehabilitation Sciences, McMaster University, Hamilton, Ontario, Canada; b School of Rehabilitation, Université de Montréal, Montréal, Quebec, Canada; c School of Rehabilitation, Faculty of Medicine and Health Sciences, Université de Sherbrooke, Sherbrooke, Quebec, Canada; d Clinical Research of the Centre Hospitalier Universitaire de Sherbrooke (CRCHUS), Sherbrooke, Quebec, Canada; e Division of Occupational and Environmental Medicine, Departments of Medicine and Public Health Sciences, University of Connecticut Health Center, Farmington, CT; f Institut de recherche Robert-Sauvé en santé et en sécurité du travail (IRSST), Montreal, Quebec, Canada; g Centre for Interdisciplinary Research in Rehabilitation of Greater Montreal (CRIR), Institut universitaire sur la réadaptation en déficience physique de Montréal (IURDPM), Centre intégré universitaire de santé et de services sociaux du Centre-Sud-de-l’Ile-de-Montréal (CCSMTL), Montreal, Quebec, Canada; h Department of Anesthesiology and Pain Medicine, Université de Montréal, Montreal, Quebec, Canada; i Research Center of the Centre Hospitalier de l’Université de Montréal, Montreal, Quebec, Canada

**Keywords:** Chronic low back pain, pain phenotyping, latent class analysis, workers, STarT Back

## Abstract

**Background**: Chronic low back pain (CLBP) is a major cause of disability globally. Stratified care has been proposed as a means to improve prognosis and treatment but is generally based on limited aspects of pain, including biopsychosocial drivers.

**Aims**: Following Initiative on Methods, Measurement, and Pain Assessment in Clinical Trials (IMMPACT) recommendations, the present study explored pain phenotypes with a sample of workers with CLBP, a population for which no pain phenotypes have been derived to date.

**Methods**: A cross-sectional design was used with a sample of 154 workers with CLBP attending a rehabilitation clinic, recruited in person and from social media. Latent class analysis was used to identify subgroups of patients with different pain profiles based on ten pain indicators (pain variability, pain intensity, pain quality, somatization, sleep quality, depression, fatigue, pain catastrophizing, neuropathic pain, and central sensitization).

**Results**: The majority of the sample (85%) were recruited through social media. Both the two-class and three-class solutions were found to be satisfactory in distinguishing phenotypes of workers with CLBP. Three variables proved particularly important in distinguishing between the pain phenotypes—pain quality, fatigue, and central sensitization—with higher scores on these indicators associated with pain phenotypes with higher pain burden. Increased chronic pain self-efficacy, work-related support, and perceived work abilities were protective risk factors for being in a higher pain burden class.

**Conclusions**: The present study is the first to explore IMMPACT recommendations for pain phenotyping with workers with CLBP. Future prospective research will be needed to validate the proposed pain phenotypes.

## RÉSUMÉ

**Contexte:** La lombalgie chronique est une cause majeure d’invalidité dans le monde. Les soins stratifiés ont été proposés comme un moyen d’améliorer le pronostic et le traitement, mais reposent généralement sur des aspects limités de la douleur, y compris des facteurs biopsychosociaux.

**Objectifs:** Suite à l’initiative sur les méthodes, la mesure et l’évaluation de la douleur dans les essais cliniques (IMMPACT), la présente étude a exploré les phénotypes de la douleur à l’aide d’un échantillon de travailleurs atteints de lombalgie chronique, une population pour laquelle aucun phénotype de douleur n’a été déterminé à ce jour.

**Méthodes:** Un devis transversal a été utilisé avec un échantillon de 154 travailleurs atteints de lombalgie chronique fréquentant une clinique de réadaptation, recrutés en personne et sur les réseaux sociaux. L’analyse des classes latentes a été utilisée pour déterminer des sous-groupes de patients présentant différents profils de douleur en fonction de dix indicateurs de douleur (variabilité de la douleur, intensité de la douleur, qualité de la douleur, somatisation, qualité du sommeil, dépression, fatigue, catastrophisation de la douleur, douleur neuropathique et sensibilisation centrale).

**Résultats:** La majeure partie de l’échantillon (85 %) a été recrutée par le biais des médias sociaux. Les solutions à deux classes et à trois classes se sont révélées satisfaisantes pour distinguer les phénotypes des travailleurs atteints de lombalgie chronique. Trois variables se sont avérées particulièrement importantes pour distinguer les phénotypes de la douleur – la qualité de la douleur, la fatigue et la sensibilisation centrale - avec des scores plus élevés pour les indicateurs associés à des phénotypes de douleur avec un fardeau de douleur plus élevé. L’augmentation de l’auto-efficacité relativement à la douleur chronique, le soutien lié au travail et les capacités de travail perçues étaient des facteurs de protection pour la classe du fardeau de douleur plus élevé.

**Conclusions:** La présente étude est la première à explorer les recommandations IMMPACT pour le phénotypage de la douleur chez des travailleurs atteints de lombalgie chronique. Des recherches prospectives futures seront nécessaires pour valider les phénotypes de douleur proposés.

## Introduction

Low back pain (LBP) is the leading cause of disability globally.^1^ In Canada, direct health care costs associated with LBP represent one-third of the costs of all musculoskeletal disorders.^2^ In Ontario, the direct health care costs of spinal-related care was estimated at CA$264 million.^3^ Accounting for 22% of all chronic pain, the low back is the most frequent pain site, more than double any other body area.^4^ In Quebec, according to statistics of the Commission des Normes et de l’Équité en Santé et Sécurité du travail,^5^ the low back is the most frequently injured body region, totaling about 25% of all occupational new cases annually. The average duration of absence from work ranges from 51 to 177 days, varying with severity of symptoms.^6–8^ Up to 90% of these workers return to work (RTW) within 3 months. However, approximately 5% of patients with LBP (those with chronic pain) incur 75% of the costs in regards to health care and societal burden, two-thirds of which are linked to indirect costs associated with loss of productivity and wages.^9^ Despite the adoption of multidisciplinary rehabilitation approaches, treatment effect sizes for this population are moderate at best.^10,11^ Thus, new strategies are needed to more effectively manage the ongoing burden of chronic LBP (CLBP).

Stratified care has long been proposed as a means to improve prognosis or treatment and to minimize associated health care costs by subgrouping patients according to key clinical characteristics.^12^ Recently, recommendations for stratification have been incorporated into clinical guidelines and recommendations for research on CLBP.^13,14^ This includes recommendations for prognostic stratification to guide therapeutic interventions and a need to further understand the relationship between outcome scores and RTW.^13^ Yet, the stratified care currently endorsed is limited and does not reflect the overall biopsychosocial domains driving pain and disability.^15^ Accordingly, the current subgroupings remain limited. An innovative approach to further refine this knowledge consists of pain phenotyping (PP).

PP is defined by the Initiative on Methods, Measurement, and Pain Assessment in Clinical Trials (IMMPACT) Group as “the ensemble of observable characteristics displayed by an organism, 4” focused on patient self-reported characteristics (e.g., psychosocial functioning), patient-reported symptoms (e.g., sleep disruption), and verbal or behavioral responses to standardized psychophysical tests of pain sensitization. The aim of identifying PP categories is to target the problematic drivers of pain.^16^ PP may provide insights into the etiology of the factors driving pain and disability and enhance our classification based on its mechanisms (nociceptive, neuropathic, or central). Further, being able to better comprehend risk factors for the identified PPs will facilitate the decision-making process to establish prognosis and inform tailored, more efficient treatment in relation to multiple people’s characteristics.^17^

Though several studies have determined clinical phenotypes in people with LBP,^18–20^ few have studied workers,^21,22^ of which only one has studied those with CLBP.^23^ We have previously published the results of a latent class analysis of clinical phenotypes of workers with CLBP and the relationship of the classes to work status. We identified three distinct clinical phenotypes representing progressive clinical severity and important differences from acute cohorts and from noninjured workers.^23^ However, this analysis was limited by the use of historical data. Development of rigorous PPs for this population could lead to improved prognosis and personalized care by tailoring treatment based on a person’s PP and may identify associations with work-specific factors and outcomes. This in turn may lead to a reduction in expenditures for work-related musculoskeletal injury. To the best of our knowledge, the present multicenter study is the first to incorporate the comprehensive IMMPACT recommendations for pain phenotyping in this population.

### Objectives

The objectives of the present study were to (1) explore and identify PPs of workers with CLBP according to IMMPACT recommendations and propose hypotheses regarding sociodemographic, work-related, and clinical risk factors of the identified PPs and (2) determine the coherence between the PPs identified and the classification from the STarT (subgroup targeted treatment) back risk assessment tool.^24^

We first anticipate identifying PPs that are distinct from each other, yet homogeneous within themselves, indicative of varying pain experiences and having the potential to highlight important indicator variables within a group. Second, based on existing evidence, we expect PPs demonstrating a greater overall pain burden to be comprised of people with greater central sensitization, poorer psychosocial status and sleep quality, and greater somatization, pain intensity, and fatigue.^25–29^ We further anticipate that the PPs will differ in their risk factors (e.g., male sex, comorbidities). Finally, we expect a moderate level of coherence between the present PPs and the STarT back risk assessment tool, which classifies people into low-, moderate-, and high-risk groups for persisting disability.^24^

### Materials and Methods

#### Study Design and Sample

The present study used a cross-sectional design. People with a diagnosis of CLBP were recruited from a network of private rehabilitation clinics providing work rehabilitation in this specific population. To be included, people had to be workers with CLBP, with or without a workers’ compensation claim and starting rehabilitation treatment. CLBP was defined as pain persisting for more than 3 months and located between the lower and posterior margin of the rib cage and the horizontal gluteal fold with or without the presence of leg pain. Exclusion criteria were low back surgery in the past year, scheduled for low back surgery, serious spinal pathology (cancer, inflammatory arthropathy), diagnosed neurological disease, pregnancy, and incapacity to complete questionnaires due to physical or mental inability.

#### Procedure and Consent

Potential participants were identified at their initial consultation in the participating clinics (via support staff and/or treating physical therapist) or through social media. Potential participants were approached by clinic staff for initial consent to be contacted by the study’s research team. A research assistant from the study called consecutive participants to screen for inclusion and exclusion criteria, explained the study and its methods, and sought verbal consent for study participation. For social media, Facebook adds were published in many private groups like classified ads, retail groups, and job and candidate search groups in Montreal and Sherbrooke areas. Interested persons had to reach out by phone or e-mail the research assistant, who then screened for their admissibility and provided all study information as well as the link for the online consent form and questionnaire. Detailed consent information was provided electronically as part of the self-reported electronic questionnaire. All participants provided written informed consent for their participation in the present study and they were informed that they would not be identifiable from the article. The research protocol and consent form were approved by the ethics committee of the Center intégré universitaire de santé et de services sociaux de l’Est-de-l’Île-de-Montréal (Ethics Registration Number: M-12-2019-1569).

### Measures

All measures were collected using an online questionnaire.

#### Class Indicators

The following indicators were measured with well-validated questionnaires whose psychometric qualities are well documented:

1. Pain variability was assessed using the images from the painDETECT questionnaire (PDQ^30,31^), which results in three categories (persistent pain with slight fluctuation, persistent pain with pain attacks, and pain attacks without pain between them).
2. Pain intensity was measured with a numeric pain rating scale on a 11-point scale inquiring about a person’s usual level of pain in the past week.^32,33^ The end points of the scale corresponding to 0 and 10 were no pain and worst possible pain, respectively.
3. Pain quality: The short-form McGill Pain Questionnaire 2^34–36^ was used to assess pain qualities. Participants were asked to qualify 22 types of pain and symptoms as experienced in the previous week on a 0 (*no pain*) to 10 (*worst possible pain*) scale with the total score used.
4. Somatization was assessed with the 15-item Physical Health Questionnaire.^37–39^ Items represent 15 somatic symptoms or symptom clusters assessed with scores of increasing severity.
5. Sleep quality was measured with the Pittsburgh Sleep Quality Index.^40,41^ The Pittsburgh Sleep Quality Index includes 19 items assessing sleep-related variables using both Likert scales and open-ended responses. Higher scores indicate worse sleep.
6. Mood and anxiety: Anxiodepressive symptoms were assessed with the Hospital Anxiety and Depression Scale.^42,43^ This measure is composed of 14 items assessing depression and anxiety symptoms answered on a 4-point scale scored 0 to 3, with higher scores indicating worse symptoms.
7. Fatigue was measured with the Multidimensional Fatigue Inventory,^44,45^ which is a 20-item self-report questionnaire scored on a 7-point scale. Higher total scores represent higher levels of fatigue.
8. Pain catastrophizing was assessed with the Pain Catastrophizing Scale,^46–49^ a 13-item instrument. Participants were asked to reflect on their past painful experiences on a 0 (*not at all*) to 4 (*all the time*) scale to what extent they experienced each of the 13 thoughts and feelings described. Higher total scores indicate worse pain catastrophizing.
9. Neuropathic pain was assessed with the self-reported PDQ.^30,31^ The PDQ is composed of questions about pain intensity, course of pain, radiation of pain and the presence and severity of seven somatosensory symptoms of neuropathic pain that are answered to on a 0 (*never*) to 5 (*very strongly*) scale.
10. Presence and severity of central sensitization pain was assessed with the Central Sensitization Inventory part A,^50,51^ which is a 25-item self-report questionnaire. Questions are answered on a 5-point scale ranging from 0 (*never*) to 4 (*always*). Higher scores represent higher severity, with a cut score of 40 indicating the presence of central sensitization.

#### Covariates

In addition to sociodemographic variables such as sex, age, height, weight, marital status, and education, the following covariates were included in the questionnaire:

1. Kinesiophobia was assessed with the Tampa Scale of Kinesiophobia-17.^52–54^ This scale is a 17-item measure assessing pain-related fear on a 4-point scale ranging from 1 (*strongly disagree*) to 4 (*strongly agree*).
2. Self-reported functional limitation was assessed with the Oswestry Disability Index.^55,56^ The Oswestry Disability Index is a ten-section measure resulting in a percentage score of the level of disability related to daily living, with higher scores representing more limited functioning. Each section represents a sphere of the person’s life, such as personal care, walking, sitting, and standing.
3. The short six-item French-Canadian Chronic Pain Self-Efficacy Scale^57^ was used to assess participants’ self-efficacy. The items are answered to on a 10-point scale ranging from 1 (*not at all confident*) to 10 (*completely confident*), with higher average scores representing higher chronic pain self-efficacy.
4. Perceived work abilities was assessed with the Work Role Functioning Questionnaire.^58,59^ The 27 items of the Work Role Functioning Questionnaire are answered to on a 5-point scale from 0 (*difficult all of the time*) to 4 (*difficult none of the time*). Higher scores represent better perceived work abilities.
5. Work-related fear was assessed with the Fear Avoidance Beliefs Work subscale.^60,61^ The Work subscale of the Fear Avoidance Beliefs questionnaire is a 16-item measure assessing fear avoidance beliefs answered on a 7-point scale ranging from 1 (*strongly disagree*) to 7 (*strongly agree*). Higher scores represent more work-related fears.
6. Perceived work-related support was assessed with the short eight-item Survey of Perceived Organizational Support.^22,62^ Items are answered on a 7-point scale ranging from 1 (*strongly disagree*) to 7 (*strongly agree*).^22,62,63^
7. The STarT back risk assessment tool^24,64^ was used to classify people into low, moderate, and high risk categories for persisting disability.
8. Finally, participants were asked whether they had any litigation with a yes/no question. Similarly, they were asked, with yes/no questions, whether they had any leg pain and whether they had a directional preference at the time of their initial consultation.

#### Data Analysis

Latent class analysis (LCA) was conducted to identify classes of individuals with different pain profiles based on ten pain indicators (see Table 1). An increasing number of class solutions starting at two were tested until the fit statistics were most satisfactory. Each class was composed of no less than 10% of the sample, and the entropy, a measure of variability where 1 indicates perfect delineation of classes, was closest to one. The fit statistics used to determine the best possible class solution were the Bayesian information criterion (BIC), the Lo-Mendell-Rubin adjusted likelihood ratio test (LMRT), and bootstrapped likelihood ratio test (BLRT). Lower values of BIC represent better fit and nonsignificant LMRT and BLRT values indicate no more significant improvement in model fit over previous models with one less class. Next, we ran logistic regression models to identify predictors of membership to the identified PPs. LCA was conducted with Mplus 6.1. SPSS v.26 was used for the logistic regression models.^87,88^

**Table 1.** Sample descriptives

Covariates	Whole sample (*n* = 154) Mean (SD)
Sex Female	58.4%
Age	39.32 (12.88)
BMI	26.8 (6.74)
Marital status Single	36.4%
Education Primary/high school/CEGEP University and higher	67.5% 32.5%
Kinesiophobia	43.21 (7.58)
Self-reported functional limitation	45.97 (13.38)
Chronic pain self-efficacy	6.37 (2.00)
Perceived work abilities	65.59 (29.75)
Work-related fear	17.70 (11.93)
STarT back Risk classification category 1–low 2–medium 3–high	55.8% 31.8% 12.3%
Perceived organizational support	28.75 (10.54)
Litigation Yes	16.7%
Leg pain Yes	57.5%
Directional preference Yes	70.1%
Method of recruitment Rehabilitation clinic Social media	15% 85%

BMI = body mass index; CEGEP = College of General and Professional Teaching.

## Results

A total of 154 people were recruited (58.4% females; 88.4% white). The average age was 39.32 (SD = 12.88, min = 18; max = 70), 36.4% were single, and 32.5% were university educated. The average body mass index was 27.80 (SD = 6.74). As mentioned before, patient recruitment was done by clinic staff or via Facebook ads in Montreal and Sherbrooke areas. For the first method, less than 15% of the participants were recruited in 1.5 years compared to more than 85% in one month by e-recruitment. Recruitment started in June 2018 and closed in March 2020. Twenty people (13%) were recruited early in the Montreal clinics and the rest of the sample (*n* = 134; 87%) was recruited in the Sherbrooke area between December 2019 and March 2020.

### Class Models

**Figure 1.**
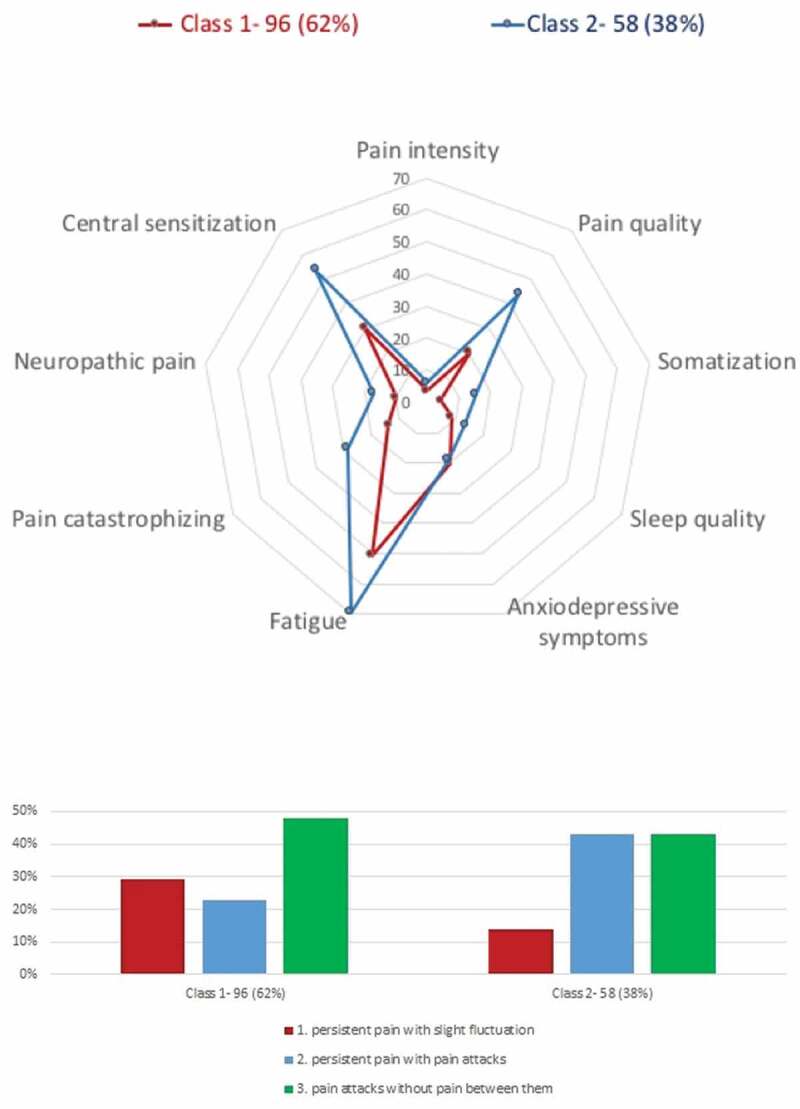
Two-class solution showing spidergram plot of means of continuous indicator variables and bar chart of proportion of categorical variable

LCA models with two, three, and four classes were tested. We stopped at model 4 because one class included less than 10% of the sample. Based on a number of fit statistics, model entropy, and sample size per class (see Table 2), we identified both the two-class and three-class models as satisfactory. Table 3 presents the descriptive data for the two-class model for each class as well as for the whole sample. Class 1 was composed of 62.3% of the sample, and it had a higher proportion of females, had a higher average age, had fewer single people, and had more university educated people than class 2. In terms of comorbidities, class 1 had lower scores of kinesiophobia, self-reported functional limitations, work-related fears, active litigation, leg pain, and directional preference than class 2. Furthermore, class 1 presented higher scores of chronic pain self-efficacy, perceived work abilities, and perceived support from their organization than class 2. Regarding class indicators (see Figure 1), class 1 presented lower mean scores on all pain indicators except for anxiodepressive symptoms, which were equivalent between classes 1 and 2. Finally, nearly half of the people in class 1 (47.9%) described their pain as “pain attacks without pain between them.” This suggests less pain burden in people in class 1 and, conversely, greater pain burden in people in class 2.

**Table 2.** Fit indices for the two-class and three-class LCA.^a^

	Two-class solution *N* = 154	Three-class solution *N* = 154
BIC	9556.45	9521.95
LMRT	431.33 (*p* < .0001)	93.26 (*p* = .1100)
BLRT	439.89 (*p* < .0001)	94.94 (*p* = .105)
Entropy	0.924	0.878
Sample size per class	C1: *n* = 96 C2: *n* = 58	C1: *n* = 64 C2: *n* = 68 C3: *n* = 22

^a^BIC: smallest value represents better fit. LMRT and BLRT: Nonsignificant *p* value represents a better fit.

LCA = latent class analysis; BIC = Bayesian information criterion; LMRT = Lo-Mendell-Rubin adjusted likelihood ratio test; BLRT = bootstrapped likelihood ratio test.

**Table 3.** class solution characteristics

	Class 1 (n = 96)	Class 2 (n = 58)
	Means (SD)	Means (SD)
**Covariates**		
Sex		
Female	60.4%	55.2%
Male	39.6%	44.8%
Age	40.21 (12.71)	37.84 (13.14)
BMI	26.66 (5.58)	29.75 (8.05)
Marital status		
Single	31.3%	44.8%
Not single	68.8%	55.25
Education		
Primary/High School/ CEGEP	59.4%	81.0%
University and higher	40.6%	19.0%
Kinesiophobia	40.53 (6.52)	47.64 (7.17)
Self-reported functional limitation	40.33 (9.32)	55.30 (13.91)
Chronic pain self-efficacy	6.91 (1.76)	5.49 (2.08)
Perceived work abilities	78.50 (21.32)	44.21 (29.53)
Work-related fear	13.92 (9.98)	23.97 (12.32)
Litigation		
Yes	12.5%	23.2%
Leg pain		
Yes	45.8%	77.2%
Directional preference		
Yes	61.5%	84.5%
Perceived organizational	31.26 (10.23)	24.60 (9.77)
support		

BMI = body mass index; CEGEP = College of General and Professional Teaching.

**Figure 2.**
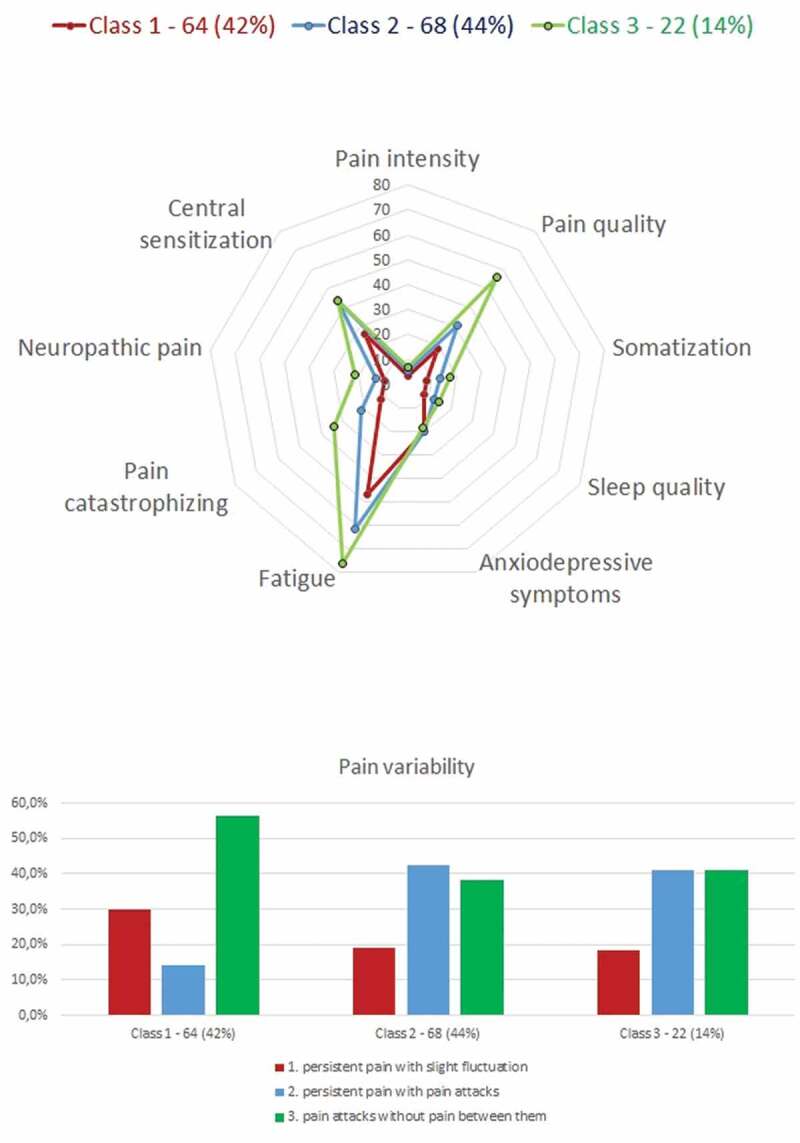
Three-class solution showing spidergram plot of means of continuous indicator variables and bar chart of proportion of categorical variable

Similarly, Table 4 presents the descriptive data for each class of the three-class model. Class 1 was composed of 41.6% of the sample (*n* = 64), class 2 was made up of 44.2% (*n* = 68), and class 3 was made up of only 14.3% (*n* = 22). Class 2 had fewer males than the other classes and class 3 had younger, more single, and fewer university-educated people than the other classes. In terms of comorbidities, people in class 1 presented the overall best profile and class 3 presented the overall worst profile. Finally, regarding the average scores on the class indicators, class 1 had the lowest average scores on every indicator except anxiodepressive symptoms (see Figure 2), which did not differ between the three classes. More than half (56%) of the people in class 1 described their pain as “pain attacks without pain between them.” Class 3 had the highest average scores on all class indicators (excluding anxiodepressive symptoms). About two in five people of both classes 2 and 3 described their pain as “persistent pain with pain attacks,” whereas only 14% of people in class 1 described their pain this way. Overall, the pain burden profile of people in class 3 was the worst, followed by people in class 2 and finally people in class 1.

**Table 4.** class solution characteristics

	Class 1	Class 2	Class 3
	(n = 64)	(n = 68)	(n = 22)
	Mean (SD)	Mean (SD)	Mean (SD)
**Covariates**			
Sex			
Female	56.3%	67.6%	40.9%
Male	43.8%	32.4%	59.1%
Age	39.75 (13.06)	40.19 (12.49)	35.36 (13.44)
BMI	26.10 (5.44)	28.20 (6.95)	31.90 (8.03)
Marital status			
Single	29.7%	38.2%	50%
Not single	70.3%	61.8%	50%
Education			
Primary/High School/ CEGEP	54.7%	73.5%%	86.4%
University and higher	45.3%	26.5%%	13.6%
Kinesiophobia	39.84 (6.05)	44.53 (7.74)	48.91 (6.58)
Self-reported functional limitation	37.05 (7.43)	49.56 (11.90)	60.82 (13.12)
Chronic pain self-efficacy	7.33 (1.63)	5.92 (1.82)	5.02 (2.32)
Perceived work abilities	84.10 (17.20)	59.60 (26.32)	30.23 (29.66)
Work-related fear	12.30 (9.02)	18.90 (11.36)	29.73 (11.60)
Litigation			
Yes	10.5%	18.5%	27.3%
Leg pain			
Yes	46.9%	58.2%	86.4%
Directional preference			
Yes	54.7%	77.9%	90.9%
Perceived organizational	33.09 (9.72)	27.26 (10.21)	20.73 (7.82)
support			

BMI = body mass index; CEGEP = College of General and Professional Teaching

### Predictors of Class Membership

Table 5 presents the results from the binary logistic regression model for the predictors of class membership for the two-class model. People in a relationship (odds ratio [OR] = 0.20, 95% confidence interval [CI], 0.06–0.76) as well as older (OR = 0.94, 95% CI, 0.80–0.99) people were less likely to be in the high pain burden class compared to those in the low pain burden class. People reporting increasing levels of functional limitation (OR = 1.10, 95% CI, 1.04–1.18) and leg pain (OR = 3.51, 95% CI, 1.05–11.05) were more likely to be in the high pain burden class than in the low one. Finally, people reporting high work abilities were less likely to be in the high pain burden class (OR = 0.97, 95% CI, 0.94–0.99) than in the low one.

**Table 5.** Predictors of class membership; two-class solution.^a^

Variable	Odds ratio	95% Confidence interval	*p* Value
Sex (male as referent)	0.29	(0.08–1.03)	.06
Age	0.94	(0.90–0.99)	.02
Marital status (not single as referent)	0.20	(0.06–0.76)	.02
Education (university educated as referent)	0.76	(0.20–2.81)	.68
Kinesiophobia	1.07	(0.98–1.18)	.13
Self-reported functional limitation	1.10	(1.04–1.18)	.003
Chronic pain self-efficacy	0.95	(0.67–1.34)	.75
Work-related fear	1.02	(0.96–1.08)	.59
Litigation (yes as referent)	0.74	(0.15–3.56)	.70
Leg pain (yes as referent)	3.51	(1.05–11.05)	.04
Directional preference (yes as referent)	2.43	(0.67–8.82)	.18
Perceived work-related support	0.97	(0.92–1.02)	.23
Perceived work abilities	0.97	(0.94–0.99)	.01

^a^Class 2 (high pain burden) as referent.

Table 6 presents the results from the multinominal logistic regression model for the predictors of class membership for the three-class model. Older people (OR = 1.26, 95% CI, 1.10–1.43) and those with higher chronic pain self-efficacy (OR = 2.07, 95% CI, 1.13–3.79), higher work-related support (OR = 1.20, 95% CI, 1.056–1.36), and higher perceived work abilities (OR = 1.11, 95% CI, 1.03–1.12) were more likely to be classified in the low pain burden class (class 1) than in the high pain burden class. However, those with increased self-reported functional limitations (OR = 0.75, 95% CI, 0.65–0.86) were less likely to be classified in the low pain burden class (class 1) than in the high pain burden class. Furthermore, older age (OR = 1.19, 95% CI, 1.06–1.33) was a predictor of being classified in the moderate pain burden class (class 2) compared to the high pain burden class. However, those with higher levels of functional limitation (OR = 0.90, 95% CI, 0.82–0.99) and work-related fear (OR = 0.90, 95% CI, 0.82–1.00) were less likely to be classified in the moderate pain burden class (class 2) compared to the high pain burden class.

**Table 6.** Predictors of class membership; three-class solution.^a^

Variable	Odds ratio	95% Confidence interval	*p* Value
Class 1 (low pain burden)			
Sex (male as referent)	0.10	(0.01–1.19)	.07
Age	1.26	(1.10–1.43)	.001
Marital status (not single as referent)	0.12	(0.01–1.14)	.07
Kinesiophobia	1.11	(0.93–1.32)	.26
Self-reported functional limitation	0.75	(0.65 –.86)	.001
Chronic pain self-efficacy	2.07	(1.13–3.79)	.02
Work-related fear	0.90	(0.79–1.02)	.10
Litigation (yes as referent)	0.42	(0.03–6.15)	.52
Perceived work-related support	1.20	(1.06–1.36)	.01
Perceived work abilities	1.09	(1.03–1.15)	.002
Class 2 (moderate pain burden)			
Sex (male as referent)	0.81	(0.11–5.71)	.83
Age	1.19	(1.06–1.33)	.003
Marital status (not single as referent)	0.49	(0.09–2.68)	.41
Kinesiophobia	1.04	(0.91–1.19)	.57
Self-reported functional limitation	0.90	(0.82–0.99)	.02
Chronic pain self-efficacy	1.04	(0.69–1.56)	.86
Work-related fear	0.90	(0.82–1.00)	.05
Litigation (yes as referent)	0.47	(0.07–3.11)	.43
Perceived work-related support	1.11	(0.99–1.24)	.07
Perceived work abilities	1.02	(0.98–1.06)	.37

^a^Class 3 (highest pain burden) as referent. Directional preference, leg pain, and education were excluded from this analysis because one category contained three or fewer individuals from class 3.

### Relationship to the STarT Back Classification

Table 7 presents the coherence between the two-class model and two categories from the (low vs. high) STarT back classification as well as the three-class model and the thee categories (low, medium, and high) STarT back classification. Almost three quarters of the people (74%) were classified similarly by the two-class model and the two-category STarT back classification (low/high). Slightly more than 60% of the people were classified similarly by the three-class model and the three-category STarT back classification.

**Table 7.** Coherence between STarT back classification and the two- and three-class solutions

	STarT back classification			
Two-class solution	Low		Medium–high	
Class 1–low	46.1%		16.2%	
Class 2–high	9.7%		27.9%	
χ^2^ (df = 1) = 33.92, *p* = .001				
Three-class solution	Low	Medium		High
Class 1–low	35.1%	5.8%		0.6%
Class 2–medium	20.1%	19.5%		4.5%
Class 3–high	0.6%	6.5%		7.1%
χ^2^ (df = 4) = 63.38, *p* = .001				

## Discussion

The present exploratory study is the first attempt at incorporating the comprehensive IMMPACT recommendations for PP in the CLBP population. PP appears to offer a promising avenue for prognosis and the development of tailored treatments to specific patient groups. Better understanding the PPs of workers is highly relevant because work-related LBP injuries are frequent and costly.^4,6,8^ In certain occupations, the incidence of LBP is easily predicted by the length of employment,^65^ suggesting that most workers in these fields will need LBP treatment over the course of their career. The first objective was to identify PPs of workers with CLBP according to IMMPACT recommendations^2^ and to determine the relationships between the identified PPs and people’s sociodemographic, work-related, and clinical risk factors. Both the two-class and three-class solutions proved to be satisfactory, with the former one showing slightly better fit and higher delineation of classes (entropy). A total of ten class indicators were studied and, as depicted in the figures, most were distinguishable between the PPs; with higher scores associated with PPs with higher pain burden.

Interestingly, anxiodepressive symptoms were not found to vary between the PPs identified in the present study. This result is surprising given the previously reported positive correlation between anxiodepressive symptoms and LBP intensity^66^ and a recent literature review that supported the claims that higher levels of anxiodepressive symptoms were predictive of worse LBP outcomes at follow-up.^67^ However, as anticipated, the present results suggest that the PPs differ in their risk factors to pain burden, with factors such as marital status, age, functional limitations, and leg pain being associated with pain burden class membership. A number of psychosocial work-related risk factors were found to be significant predictors of class membership. Specifically, the present results indicated that high levels of perceived work-related support and perceived work abilities as well as lower work-related fear were predictors of membership to lower pain burden classes. This is in line with a recent meta-analysis^68^ that reported significant relationships between psychosocial work factors and CLBP. Specifically, workload, decision authority, job control, and social support were found to be significant predictors of the presence of CLBP. Others have also reported predictive relationships between work-related factors such as self-efficacy for RTW^69–74^ and work-related fear avoidance beliefs^75–80^ and work status in people with LBP. The results support the importance of psychosocial work variables in pain burden classifications. Psychosocial work-related variables should thus be considered when managing workers with CLBP.

The second objective of the present study was to determine the coherence between the PPs identified with the present sample and the STarT back classification.^24^ The STarT back tool classifies people into low, moderate, and high risk categories for persisting disability based on a prognostic screening. Research has shown that, when proper treatment is implemented based on the STarT back classification, it leads to improvements in outcomes as well as to lower health care–related costs.^81,82^ However, some recent evidence suggests that its prognostic ability can be limited.^83,84^ Furthermore, a recent semi-systematic review suggested that other tools (e.g., Orebro Musculoskeletal Pain Screening Questionnaire) may be better at predicting work absenteeism than the STarT back tool.^85^ We thus expected a moderate level of coherence between the PPs identified with the present sample and the classification of the same people based on the STarT back tool. With the two-class solution, a 74% coherence was found, with more people with medium to high risk classified in the lower pain burden PP than people with low risk classified in the greater pain burden PP. With the three-class solution, 61.7% of people were similarly classified. The largest difference was found among those in the medium pain intensity PP. Specifically, there were as many people in this PP classified as low risk as there were people classified as medium risk. These results suggest that some of the variability in patient classification may be captured by the present PPs, which are based on a larger set of biopsychosocial characteristics.

### Limitations and Clinical Relevance

The present study has several limitations. First, the sample size was relatively small and the results need to be replicated in larger studies. Second, the study design was a cross-sectional one. Future research requires the use of a prospective outcome to better detect the nuances in pain phenotyping and its relationship with work-related outcomes. In addition, the difference in rates of recruitment favoring social media versus in person may have influenced our results. The effects of this difference could be a source of selection bias. For example, the percentage of females in our sample may be higher than typically seen in this population.^86^ The proposed PPs present an interesting option for professionals to better guide their management of workers’ CLBP; however, the present study is exploratory and will require further validation.

## Conclusion

Pain phenotyping can be used to establish prognosis and inform tailored treatment in relation to people’s multiple characteristics. The present study is the first to incorporate the comprehensive IMMPACT recommendations for pain phenotyping with a population of workers. Informative PPs were identified and a number of covariates were explored. Overall, of the pain-related biopsychosocial variables explored for class identification, three proved particularly important in distinguishing between the PPs: (1) pain quality, (2) fatigue, and (3) central sensitization, with higher scores on these indicators associated with PPs with greater pain sensitivity. Future prospective research will be needed in order to validate the present findings.
